# Selective Radionuclide Localisation in Primary Liver Tumours

**DOI:** 10.1155/1994/93101

**Published:** 1994

**Authors:** J. R. Novell, A. J. Green, A. J. W. Hilson, G. Dusheiko, R. Dick, K. E. F. Hobbs

**Affiliations:** University Dept of Surgery Depts of Nuclear Medicine and Radiology Clinical Oncology Unit Royal Free Hospital London UK

## Abstract

The therapeutic potential of ^131^I-Lipiodol was investigated in 8 patients with cholangiocarcinoma (CCA) and 15 patients with hepatocellullar carcinoma (HCC). Patients received one or two doses of ^131^I-Lipiodol via hepatic arterial injection. The mean total administered activity was 668 (SD 325) MBq in CCA and 953 (SD 477) MBq in HCC. One patient with CCA retained ^131^I-Lipiodol. The cumulative radiation dose was 9.6 Gy to tumour, 6.4 Gy to liver and 1.5 Gy to lung. The patient remained asymptomatic with no evidence of tumour 30 months from the start of treatment, whereas the remaining 7 patients exhibited tumour progression. The mean survival in CCA was 11.6 (SD 14.5) months. All 15 patients with HCC retained ^131^I with tumour: liver ratios of up to 30:1. The mean cumulative radiation dose was 34.7 (SD 32.4) Gy to tumour, 3.3 (SD 1.5) Gy to liver and 4.4 (SD 2.3) Gy to lung. The mean dose per administered activity was 3.8 (SD 4.1) cGy/MBq. Partial response (reduction in tumour size > 50%) was observed in 6 patients (40%). The mean survival was 7.1 (SD 6.0) months.

^131^I-Lipiodol can deliver highly selective internal irradiation to foci of HCC with evidence of objective response and may be the treatment of choice for patients with cirrhosis and a small tumour.


HPB Surgery, 1994, Vol. 7, pp. 185–200
Reprints available directly from the publisher
Photocopying permitted by license only

© 1994 Harwood Academic Publishers GmbH
Printed in the United States of America

### SELECTIVE RADIONUCLIDE LOCALISATION IN PRIMARY LIVER TUMOURS

### (PILOT STUDY)

J.R. NOVELL, A.J. GREEN, A.J.W. HILSON, G. DUSHEIKO, R. DICK and K.E.F. HOBBS

*University Dept of Surgery, Depts of Nuclear Medicine and Radiology, and Clinical Oncology Unit, Royal Free Hospital, London, UK*

*(Received 8 February 1993)*

The therapeutic potential of $^{131}$I-Lipiodol was investigated in 8 patients with cholangiocarcinoma (CCA) and 15 patients with hepatocellullar carcinoma (HCC). Patients received one or two doses of $^{131}$I-Lipiodol via hepatic arterial injection. The mean total administered activity was 668 (SD 325) MBq in CCA and 953 (SD 477) MBq in HCC. One patient with CCA retained $^{131}$I-Lipiodol. The cumulative radiation dose was 9.6 Gy to tumour, 6.4 Gy to liver and 1.5 Gy to lung. The patient remained asymptomatic with no evidence of tumour 30 months from the start of treatment, whereas the remaining 7 patients exhibited tumour progression. The mean survival in CCA was 11.6 (SD 14.5) months. All 15 patients with HCC retained $^{131}$I with tumour: liver ratios of up to 30:1. The mean cumulative radiation dose was 34.7 (SD 32.4) Gy to tumour, 3.3 (SD 1.5) Gy to liver and 4.4 (SD 2.3) Gy to lung. The mean dose per administered activity was 3.8 (SD 4.1) cGy/MBq. Partial response (reduction in tumour size > 50%) was observed in 6 patients (40%). The mean survival was 7.1 (SD 6.0) months.

$^{131}$I-Lipiodol can deliver highly selective internal irradiation to foci of HCC with evidence of objective response and may be the treatment of choice for patients with cirrhosis and a small tumour.

KEY WORDS: Hepatocellular carcinoma, cholangiocarcinoma, $^{131}$I-Lipiodol

### INTRODUCTION

Lipiodol (Lipiodol Ultra Fluid, May & Baker, England) is a lipid derived from poppyseed oil. It contains 475mg of iodine per ml (38% by weight) and has been used for many years as a contrast medium, principally in lymphography. In 1979 Nakakuma *et al.* injected Lipiodol into the hepatic artery and demonstrated its selective retention in foci of hepatocellular carcinoma[1]. Using a simple exchange reaction[2], part of the iodine component of Lipiodol may be replaced by the radioactive isotope $^{131}$I, making it an ideal vehicle for the delivery of internal radiotherapy, $^{131}$I-Lipiodol has been studied using scintigraphy in human subjects and exhibits a similar pattern of uptake and retention to the non-radioactive lipid[3–6]. Tsai *et al.* have measured the uptake of $^{131}$I-Lipiodol in an animal tumour model and have shown it to have a significantly longer effective half life in tumour than in normal tissue, resulting in almost complete necrosis of the tumour[7]. Toxicity to thyroid, bone marrow or lung has not been reported.

This paper is based on a presentation to the 4th World Congress of HPB Surgery in Hong Kong, June 1992.

Previous authors have reported promising results using $^{131}$I-Lipiodol in hepatocellular carcinoma (HCC), with tumour shrinkage and symptomatic improvement in the majority of patients[5,8]. Response has been reported to be greatest in small foci of HCC without significant vascular shunting[9]. In a prospective analysis of fifty patients with Stage I and II HCC by Le Jeune *et al.* the overall survival at 6 months, 12 months and 2 years was 60%, 31% and 23% respectively[10]. Initial studies have shown $^{131}$I retention in small colorectal metastases, but localisation in larger deposits is very poor[11].

Our earlier studies on Lipiodol-targeted chemotherapy[12] showed no evidence of response in the tumours studied, although survival in Stage I and II HCC was better than expected. They did, however, demonstrate excellent deposition and retention of Lipiodol in HCC, particularly smaller tumour foci. The aim of this study was to investigate the therapeutic potential of $^{131}$I-Lipiodol in two histological variants of primary liver cancer.

### PATIENTS, MATERIALS AND METHODS

### *Patients*

Inclusion criteria for the trial were unresectable cholangiocarcinoma or hepatocellular carcinoma.

### *Cholangiocarcinoma (CCA)*

All patients had unresectable intrahepatic or hilar tumours. Exclusion criteria were as follows:

— Patients older than 75 yrs.
— Serious concurrent medical conditions.
— WHO Performance Grade $> 2$
— Haemoglobin $< 10$ g/dl.
— Severe clotting abnormalities.
— Hepatic failure.
— Extrahepatic metastases.

The treated group comprised eight patients with a median age of 56 years (range 44–62 years). There were five males and three females. Histological proof of the diagnosis was obtained in six patients; in the remaining two cases (Patient Nos. 04 & 07) endoscopic biopsy, biliary cytology and in one case open biopsy at laparotomy were persistently negative. In these cases the diagnosis of CCA was established on the basis of the following criteria:

(i) a discrete stricture of the hilar or intrahepatic bile ducts;
(ii) a corresponding low-attenuation area on CT which did not demonstrate uniform vascular enhancement following intravenous injection of contrast media;
(iii) a hypovascular appearance on angiography, with or without vascular encasement. Prior to $^{131}$-Lipiodol therapy all patients underwent percutaneous insertion of a biliary endoprosthesis, with good symptomatic relief in all cases.

*Hepatocellular carcinoma (HCC)*

Exclusion criteria were as for CCA, with the addition of Okuda Stage III tumours. The treated patients with HCC comprised 14 caucasians and one negro. There were 13 men and 2 women, with a median age of 64 years (range 41–75 years). A histological diagnosis of hepatocellular carcinoma was obtained in 13 patients. In two patients with a negative biopsy (Patient Nos. 14 & 21) a hypervascular appearance at angiography in combination with a raised serum alpha fetoprotein (AFP) was regarded as diagnostic of HCC. In 12 patients the tumour occurred in a cirrhotic liver; three patients had no clinical or histological evidence of cirrhosis, including one with recurrent fibrolamellar HCC. Tumours were staged according to the classification of Okuda[13]. Four patients had Stage I tumours at diagnosis (including two with newly-diagnosed recurrence following hepatic resection) and 11 patients had Stage II tumours.

*Technique*

Seldinger catheterisation of the hepatic artery under fluoroscopic control was performed via a femoral artery puncture. 15–40 mCi $^{131}$I-radiolabelled Lipiodol (CIS Bioindustries, Saclay, France), was diluted to a total volume of 12ml with cold Lipiodol using two sterile 20ml glass syringes and a 3-way tap with luer locks. This volume of Lipiodol was administered by slow injection into the hepatic artery and the system flushed with 20ml of normal saline. Firm pressure was applied to the groin puncture site for 5 mins using a sterile lead-gloved hand.

All procedures were designed to keep radiation exposures to staff as low as reasonably achievable. Catheterisations were performed at the end of a session in the Angiography suite to allow time for decontamination should any spillage occur, and only essential staff were present in the room during the procedure. Glass syringes were employed throughout as Lipiodol has a solvent action on polystyrene. Monitoring of both room and staff was carried out immediately following completion of the procedure.

Routine clinical observations were carried out following angiography. Daily haematological and liver indices were performed for the duration of the inpatient stay. The treatment was repeated in patients who showed continued clinical wellbeing and/or reduction in tumour size. The median administered activity was 600 MGq (range 300–1160 MBq) in CCA and 875 MBq (range 525–2055 MBq) in HCC.

*Tumour Assessment*

All patients underwent $^{99}$Tc colloid liver scintigraphy prior to treatment for comparison with later $^{131}$I scintigraphy. To assess Lipiodol uptake and response to treatment, a CT scan of the liver was performed at 10 days. Perpendicular diameters were measured for each tumour focus. Tumour volumes were calculated from the CT scan using the formula:

$$\text{Volume} = \text{maximum tumour diameter} \times \text{perpendicular diameter}^2$$

Tumour response was assessed on lesion size (determined on serial CT measurements) and tumour marker levels. Patients were graded Complete Response (CR),

Partial Response (PR), No Change (NC) or Progressive Disease (PD) according to WHO criteria[14]. Toxicity grading was also assessed on WHO criteria[14]. AFP levels were measured at diagnosis and during treatment; they were within normal limits in all patients with CCA.

Liver retention of the labelled lipid was assessed by scintigraphy at 24–48 hours and 6–8 days using a Scintronix 480S Digicamera fitted with a high energy 1.9mm hexagonal-holed plane collimator. Planar anterior and posterior images of the liver and thorax were acquired over a five minute period and stored on floppy disk for later analysis. The pattern of uptake was compared to the distribution of Lipiodol within tumour tissue on synchronous CT scans of the liver and to $^{99}$Tc uptake on the pre-treatment colloid scan. Using the acquired images, regions of interest were drawn around the tumour, liver and lungs, and the total counts recorded for each region. Counts were also recorded over an equivalent area of low activity distant from the liver, and this "background" count subtracted from the tissue count in each case.

Three patients with HCC were scanned on an IGE Gemini 700 camera fitted with a 400KeV parallel-hole high resolution collimator, linked to a Saturn nuclear medicine computer for data acquisition and image processing. IGE software was used for tomographic image reconstruction and dosimetry. The doses of radioactivity administered to the tumour, surrounding liver and lung were calculated using a standard formulae[15,16].

### RESULTS

### *Cholangiocarcinoma*

#### *Localisation*

Although prolonged retention of activity was observed in the peripheral liver parenchyma in patients with hilar tumours, a comparison of the scintigraphic and tomographic appearances 6–10 days after treatment showed no evidence of tumour-selective retention of the radio-isotope in any patient. However, diffuse retention was observed in one patient (Patient No. 07) who was reassessed 7 weeks following treatment and found to retain focal activity in the hilar mass (Figure 1). She has received a further course of $^{131}$I-Lipiodol and remains alive and well 30 months from the start of treatment. The cumulative radiation dose from two treatments was calculated to be 9.6 Gy to tumour, 6.4 Gy to liver and 1.5 Gy to lung.

#### *Outcome*

The clinical details and results of treatment in CCA are summarised in Table 1. The median survival from $^{131}$I-Lipiodol treatment was 4.0 months (range 1.0–35.5 months). Five patients died from tumour progression within 4 months of treatment. The longest surviving patient has also shown evidence of disease progression clinically and has required readmission for recurrent obstructive jaundice, treated by segment III hepatico-jejunostomy.

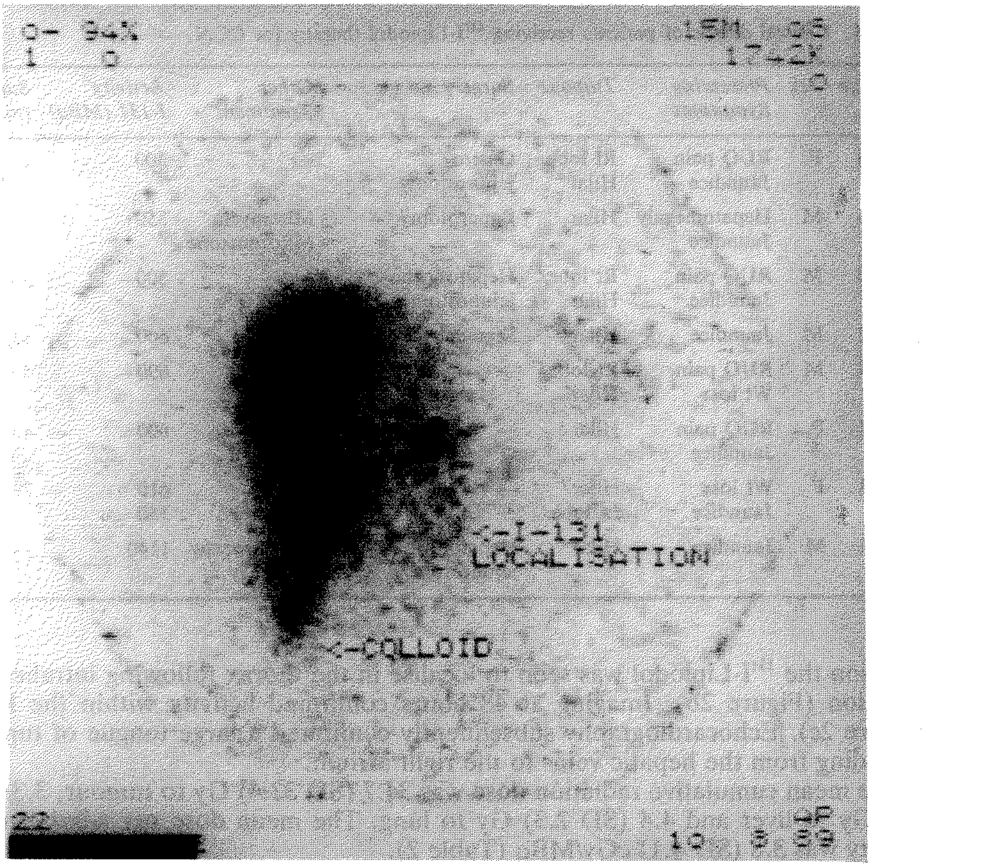

**Figure 1** Focal activity in hilar CCA at 7 weeks (AP gamma scan superimposed on colloid scan).

*Hepatocellular Carcinoma*

*Localisation*

Selective localisation of the isotope was demonstrated in all fifteen patients with tumour: liver ratios of up to 30:1. The mean tumour: liver ratio was 9:1 (SD 8). Although good localisation of the isotope was observed even in large, multifocal deposits the highest tumour: liver ratios were seen in patients with small discrete tumours. In one case tumour $^{131}I$ uptake was not confined to the liver. The patient (No. 19), a 41 year old man with a massive central hepatoma, received a small initial dose of isotope due to concern over poor liver function. Gamma imaging showed localisation within the liver but also demonstrated a small mediastinal nodule (Figure 2a). Five months later he remained well with no change in size of the tumour on CT scan and a second larger dose was administered. On this

**Table 1** Clinical details of patients receiving $^{131}$I-Lipiodol therapy for CCA

| *Pt.* | *Age* | *Sex* | *Presenting Symptoms* | *Tumour* | *Surgery* | *Other Treatment* | *Activity I-131 (MBq)* | *Survival (mnths)* |
|---|---|---|---|---|---|---|---|---|
| 01 | 44 | F | RUQ pain<br>Jaundice | Rt lobe/<br>Hilar | Gastro-<br>jejunostomy | — | 300 | 2.8 |
| 02 | 55 | M | Hepatomegaly<br>Jaundice | Hilar | Laparotomy | Mitomycin<br>Mitozantrone | 350 | 0.9 |
| 03 | 60 | M | RUQ pain<br>Jaundice | Rt lobe/<br>Hilar | Hepatodocho-<br>jejunostomy | — | 500 | 4.0 |
| 04 | 62 | M | Jaundice | Hilar | Segment III bypass | — | 600 | 35.5 |
| 05 | 58 | M | RUQ pain<br>Wt loss | Rt lobe/<br>Hilar | — | — | 690 | 4.0 |
| 06 | 57 | F | RUQ pain<br>Jaundice | Hilar | — | — | 600 | 4.0 |
| 07 | 54 | F | Wt loss<br>Jaundice | Hilar/<br>Lt lobe | — | — | 610<br>550 | 29.8 |
| 08 | 53 | M | Jaundice | Hilar | Hepatodocho-<br>jejunostomy | Radiotherapy<br>4800 cGy | 1140 | — |

occasion the $^{131}$I-Lipiodol was seen to localise in the thorax following intrahepatic injection (Figure 2b). Imaging at 48 hours confirmed activity within the heart (Figure 2c). Echocardiography subsequently confirmed a large tongue of tumour extending from the hepatic veins to the right atrium.

The mean cumulative radiation dose was 34.7 (SD 32.4) Gy to tumour, 3.3 (SD 1.5) Gy to liver and 4.4 (SD 2.3) Gy to lung. The mean dose per administered activity was 3.8 (SD 4.1) cGy/MBq (Table 2).

*Outcome*

Partial tumour response (PR) was seen in 6 patients (Table 3), with reduction in tumour size > 50% in all 6 and reduction in serum AFP levels in two. No change (NC) was observed in 4 patients and 4 patients had progressive disease (PD). Patients exhibiting PD received a significantly lower mean tumour radiation dose than patients with PR or NC (Figure 3, $p = 0.04$, pooled estimate of variance).

As we have previously reported[17], histological proof of response was obtained in one patient (No. 10) with recurrent HCC. Good tumour localisation was observed following $^{131}$I-Lipiodol administration (Figure 4a,b) and 3 months later a 5 cm wedge of liver containing 2 discrete tumour nodules was resected. Histological examination of the resected specimen showed both tumour nodules to be completely necrotic and surrounded by a zone of fibrotic but non-malignant parenchyma (Figure 4c).

Five patients were alive and under regular follow-up at the end of the trial, having survived 4–19 months from the start of treatment. Eight patients died, two from widespread extrahepatic metastases and two from hepatic failure. Four patients died from tumour progression: all had extensive multifocal disease which had failed to respond to previous chemotherapy. Two patients were lost to follow-up, one of whom was in remission at 16 months. Survival in HCC, calculated by life-table analysis, was 61% at 6 months and 31% at 1 year (Figure 5). The median

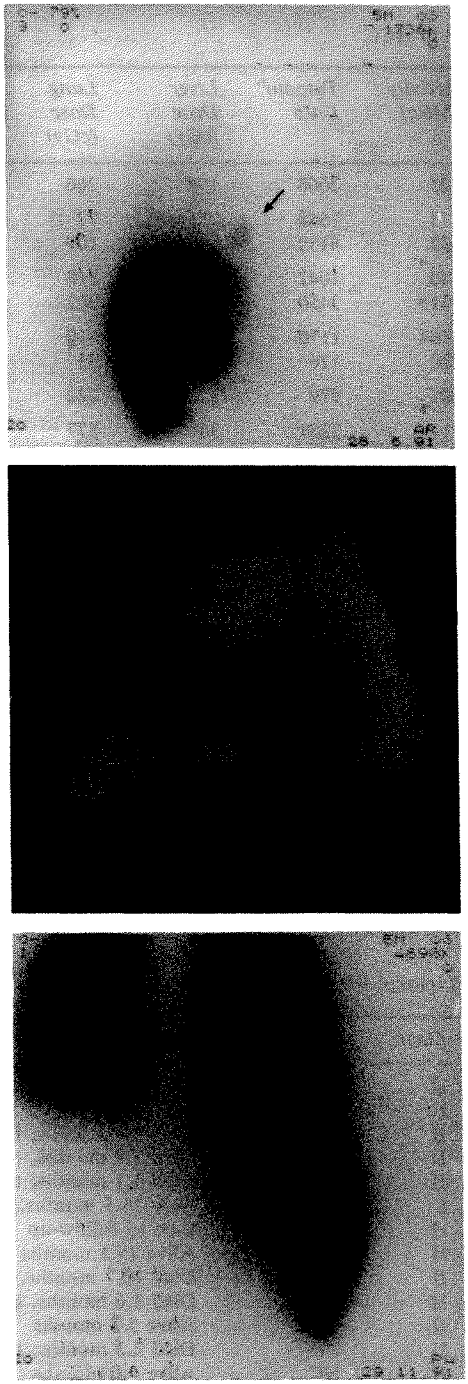

(a)

(b)

(c)

**Figure 2** Intracardiac localisation of $^{131}I$. (a) Activity in mediastinal nodule (AP gamma scan at 48 hours) (b) Supra-diaphragmatic focus of activity (Oblique lateral CXR at 1 hour) (c) Activity in heart (AP gamma scan at 48 hours).

**Table 2** Tissue Dosimetry in HCC

| *Pt No* | *Tumour Volume ($cm^3$)* | *Activity (MBq)* | *Tumour Dose* | *Liver Dose (cGy)* | *Lung Dose (cGy)* | *T/L Ratio (cGy)* | *Dose/ Activity (cGy/MBq)* |
|---|---|---|---|---|---|---|---|
| 09 | 108 | 525 | 2068 | 199 | 266 | 10.4 | 3.9 |
| 10 | 15 | 475 | 7668 | 255 | 77 | 30.0 | 16.1 |
| | 22 | 632 | 4132 | 232 | 109 | 17.8 | 6.5 |
| 11 | 422 | 740 | 1042 | 150 | 248 | 6.9 | 1.4 |
| | 212 | 1315 | 3450 | 367 | 382 | 9.4 | 2.6 |
| 12 | 632 | 1164 | 1170 | 324 | 610 | 3.6 | 1.0 |
| | 822 | 280 | 176 | 92 | 93 | 1.9 | 0.6 |
| 13 | 1255 | 601 | 579 | 322 | 222 | 1.8 | 1.0 |
| 14 | 29 | 713 | 3257 | 347 | 377 | 9.4 | 4.6 |
| 15 | 45 | 720 | 2657 | 226 | 441 | 11.8 | 3.7 |
| 16 | 436 | 800 | 713 | 262 | 405 | 2.7 | 0.9 |
| | 136 | 992 | 365 | 372 | 91 | 1.0 | 0.4 |
| 17 | 38 | 961 | 2771 | 184 | 199 | 15.0 | 2.4 |
| 18 | 1200 | 875 | 1734 | 255 | 727 | 6.8 | 2.0 |
| 19 | 1200 | 247 | 40 | 102 | 66 | 0.4 | 0.2 |
| 20 | 934 | 800 | 500 | 350 | 696 | 1.4 | 0.6 |
| 21 | 72 | 743 | 4875 | 230 | 332 | 21.2 | 7.6 |
| | | | 742 | | | | 3.2 |
| 22 | 250 | 885 | 7642 | 446 | 506 | 17.1 | 8.6 |
| 23 | 64 | 853 | 3687 | 198 | 757 | 18.6 | |
| | | | 1432 | | | 7.2 | |
| | | | 730 | | | 3.7 | 7.6 |
| | | | 635 | | | 3.2 | |

**Table 3** Results of 131-I Lipiodol treatment in HCC

| *Pt* | *Age* | *Sex* | *Stage* | *Response* | *Outcome* |
|---|---|---|---|---|---|
| 09 | 63 | M | II | PR | Died 3.0 months, bone 2° |
| 10 | 70 | F | I | PR | Lost to follow-up 16.2 months |
| 11 | 75 | M | II | PR | Died 4.0 months, hepatorenal failure |
| 12 | 68 | M | II | PD | Died 6.8 months, tumour progression |
| 13 | 69 | M | II | PD | Died 1.1 months, tumour progression |
| 14 | 64 | M | I | PR | Died 16.5 months, hepatic failure |
| 15 | 62 | M | II | — | Lost to follow-up |
| 16 | 42 | F | II | PR | Alive 19.1 months |
| 17 | 57 | M | II | PR | Died 10.7 months, tumour progression/bone 2° |
| 18 | 65 | M | II | PD | Died 3.6 months, tumour progression |
| 19 | 41 | M | II | NC | Alive 5.2 months |
| 20 | 75 | M | II | PD | Died 2.3 months, tumour progression |
| 21 | 72 | M | II | NC | Alive 4.0 months |
| 22 | 50 | M | II | NC | Alive 3.8 months |
| 23 | 64 | M | I | NC | Alive 3.8 months |

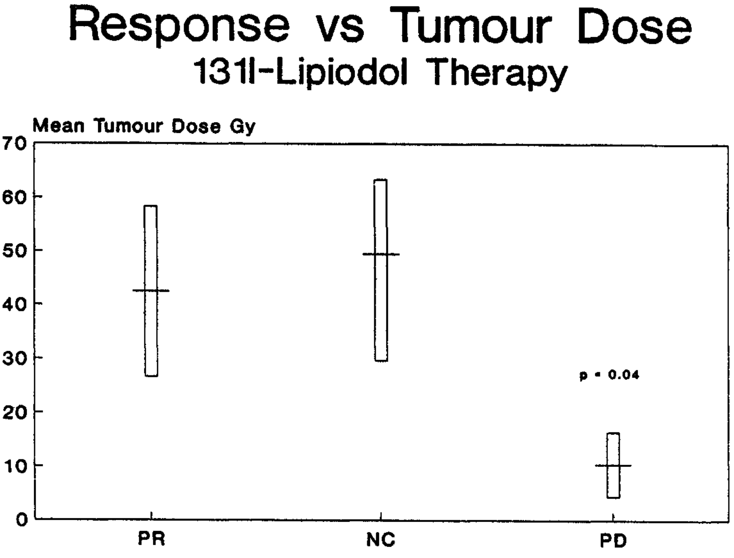

**Figure 3** Radiation dose versus response in HCC.

survival was 4.0 months (range 1.1 – 19.1 months). The median survival by Okuda stage was 16.2 months (range 3.8 – 16.5) for Stage I and 4.0 months (range 1.1 -- 19.1) for Stage II tumours.

*Adverse Effects*

There were no complications associated with percutaneous catheterisation. No significant activity was recorded in thyroid or bone marrow on whole body gamma scintigraphy, and no patient developed thyroid insufficiency or haematological evidence of marrow depression. In patients with small, discrete deposits of HCC activity in the lung fields remained low following intrahepatic injection but in patients with massive or multifocal tumours hepatopulmonary shunting of the isotope was observed. The mean radiation dose to the lungs was less than 5 Gy and no clinical signs of acute pneumonitis or chronic pulmonary fibrosis were observed in any patient. Mild to moderate pyrexia (Grade 1–2) was seen following 13 treatments (45%). Mild to moderate elevation of liver transaminases and bilirubin (Grade 1–2) occurred following 10 treatments (35%). Significant hepatotoxicity occurred in one patient (No. 11), a 75 year old cirrhotic (Child-Pugh Grade B) who developed hepatic encephalopathy and oliguria 2 days following a second treatment with $^{131}$I Lipiodol. The dose of radioactivity to tumour and non-neoplastic liver was calculated to be 34.5 Gy and 3.7 Gy respectively. Liver ultrasound demonstrated liquefaction of the tumour mass, and his deterioration in liver function was ascribed to massive tumour necrosis. He was resuscitated and showed a slow but progressive improvement in hepatorenal function. A further decline in his condition was associated with episodes of sepsis, and he died one month following the treatment.

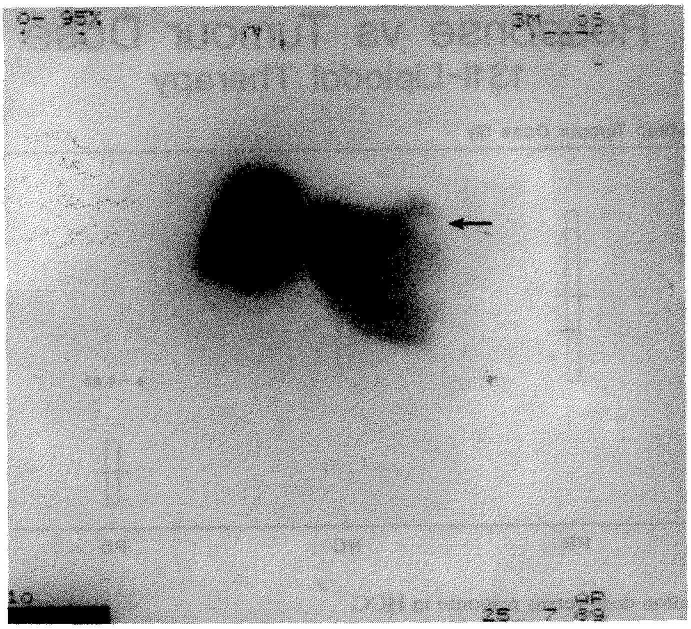

(a)

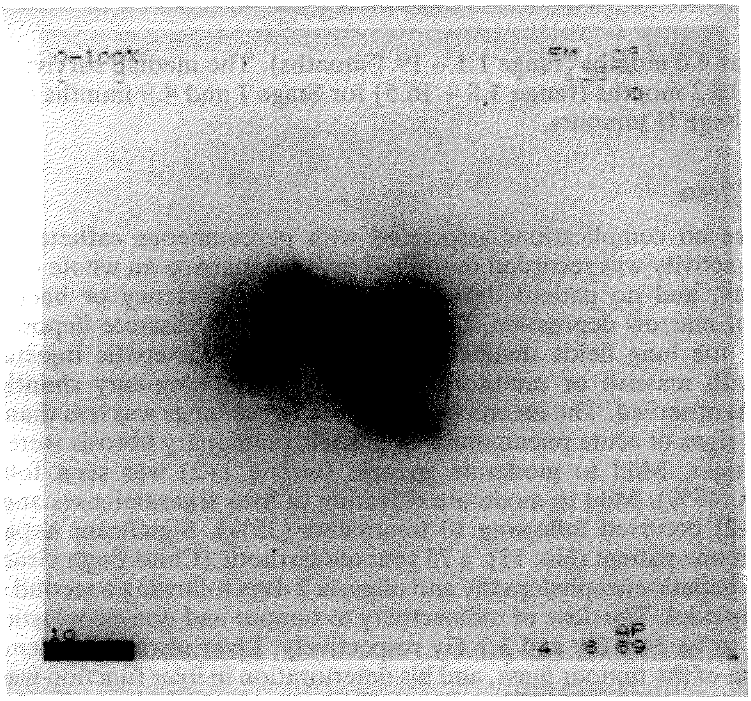

(b)

**Figure 4** Response in HCC. (a) Uptake defect due to tumour in left hepatic lobe (AP colloid scan) (b) $^{131}I$ uptake by tumour (AP gamma scan at 48 hours) (c) Light micrograph (reticulin stain) showing zone of fibrosis surrounding necrotic tumour nodule.

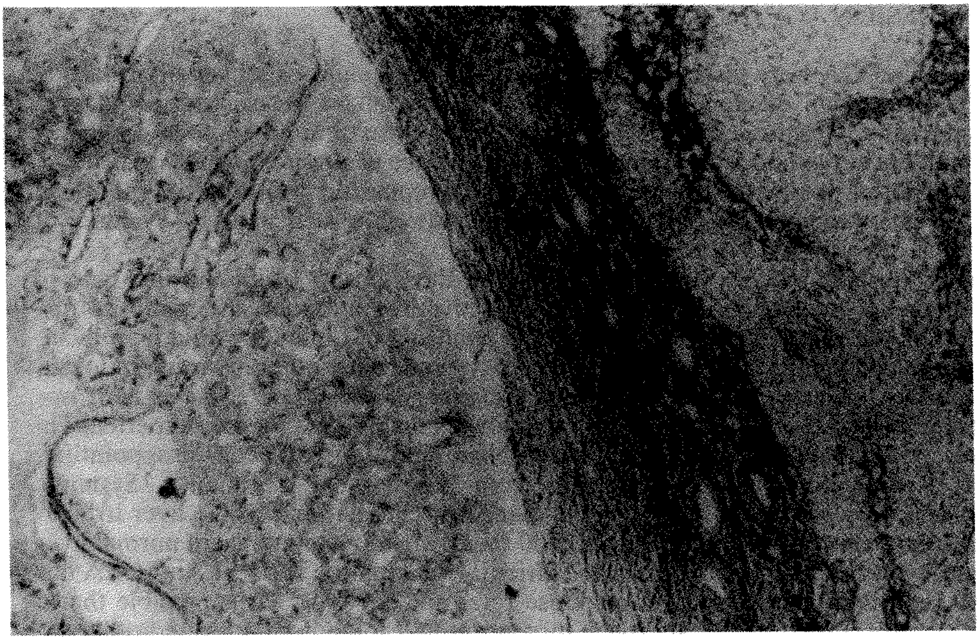

**Figure 4** (continued) (c)

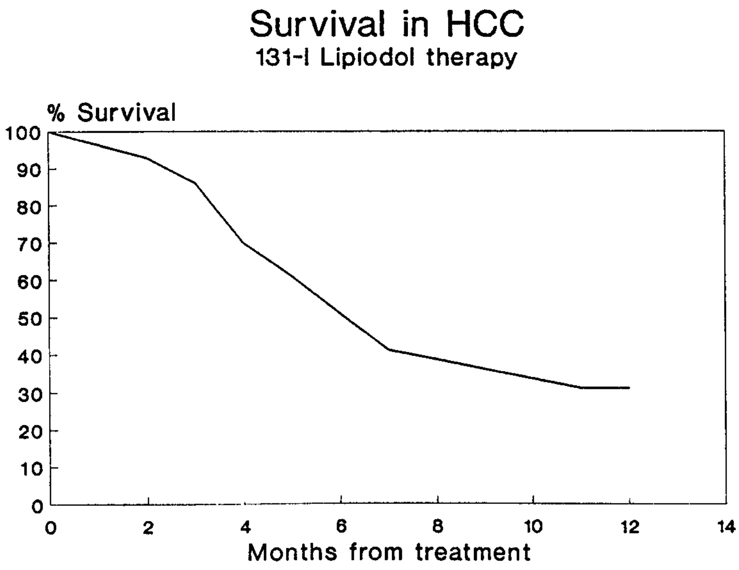

**Figure 5** Survival in HCC following $^{131}$I-Lipiodol therapy.

### DISCUSSION

Results in CCA were disappointing, with only one of eight patients demonstrating limited retention of activity in the region of the biliary obstruction on gamma scintigraphy. Despite a relatively small radiation dose of approximately 1000 cGy this patient survived for 30 months with no evidence of progression of her disease. She required two hospital admissions for stent replacement during the follow-up period but is currently asymptomatic with no indwelling stents and no evidence of residual disease on CT scan.

To date there has been only one report on the use of $^{131}$I-Lipiodol in a case of intrahepatic cholangiocarcinoma[18]: no selective retention of the radio-isotope occurred. Targeted chemotherapy using an emulsion of Lipiodol with doxorubicin, in combination with stenting and long term systemic chemotherapy using mitozantrone, has been described in one case of cholangiocarcinoma[19]. The patient was reported to be alive and well 27 months following diagnosis. Our own experience in three cases treated with a Lipiodol-epirubicin emulsion has been disappointing, with a mean survival of 5.4 months from the start of treatment[12]. Systemic chemotherapy is rarely of benefit in cholangiocarcinoma, and whole liver irradiation is dose-limited to a maximum of 3500 rads by the low tolerance of normal liver tissue. However, the use of these modalities in combination with $^{131}$I Anti-CEA has been reported to increase both response rates and survival[20]. It is possible that a similar radical multimodal approach incorporating Lipiodol-targeted radiotherapy may provide an effective means of palliation in some patients with cholangiocarcinoma.

All fifteen patients with HCC showed tumour-specific retention of activity with tumour: liver dose ratios of up to 30:1. Previous investigators have found $^{131}$I-Lipiodol to be retained almost exclusively by the liver and lungs[3]. A measure of the accuracy of the dosimetry calculations used in this study can therefore be obtained from comparison of tissue activities with the administered activity, derived from the measured activity of the $^{131}$I-Lipiodol aliquot prior to injection minus the residual activity the end of the procedure. Calculation of the combined activities in tumour, liver and lung at time $t = 0$ gave a median value of 87% of the estimated administered activity, with a range of 70 – 118%. Taking into account losses to other organs via the splanchnic circulation, and overestimation of the administered activity due to adherence of activity to the syringe and catheter, this is an acceptable accuracy for planar dosimetry. However, the accuracy of dosimetry based on planar scintigraphy is inferior to that achievable using the more sophisticated technique of SPECT imaging. For the last three cases in this series we had access to a tomographic gamma camera, which acquires 60 images at intervals of 5 degrees rotation with an exposure time of 5 seconds - 1 minute. By constructing a tomographic gamma image this effectively eliminates errors from "background" activity in surrounding tissues.

Partial tumour response was seen in two of three patients with Stage I tumours (67%) and four of eleven patients with Stage II tumours (36%). There was no significant difference in response rates between Stage I and II tumours ($p = 0.76$, Fisher exact probability test).

Patients showing response included one patient with fibrolamellar HCC. The patient (No. 16), a 42 year old woman, presented with a massive tumour of the right hepatic lobe invading the diaphragm, which had failed to respond to intra-

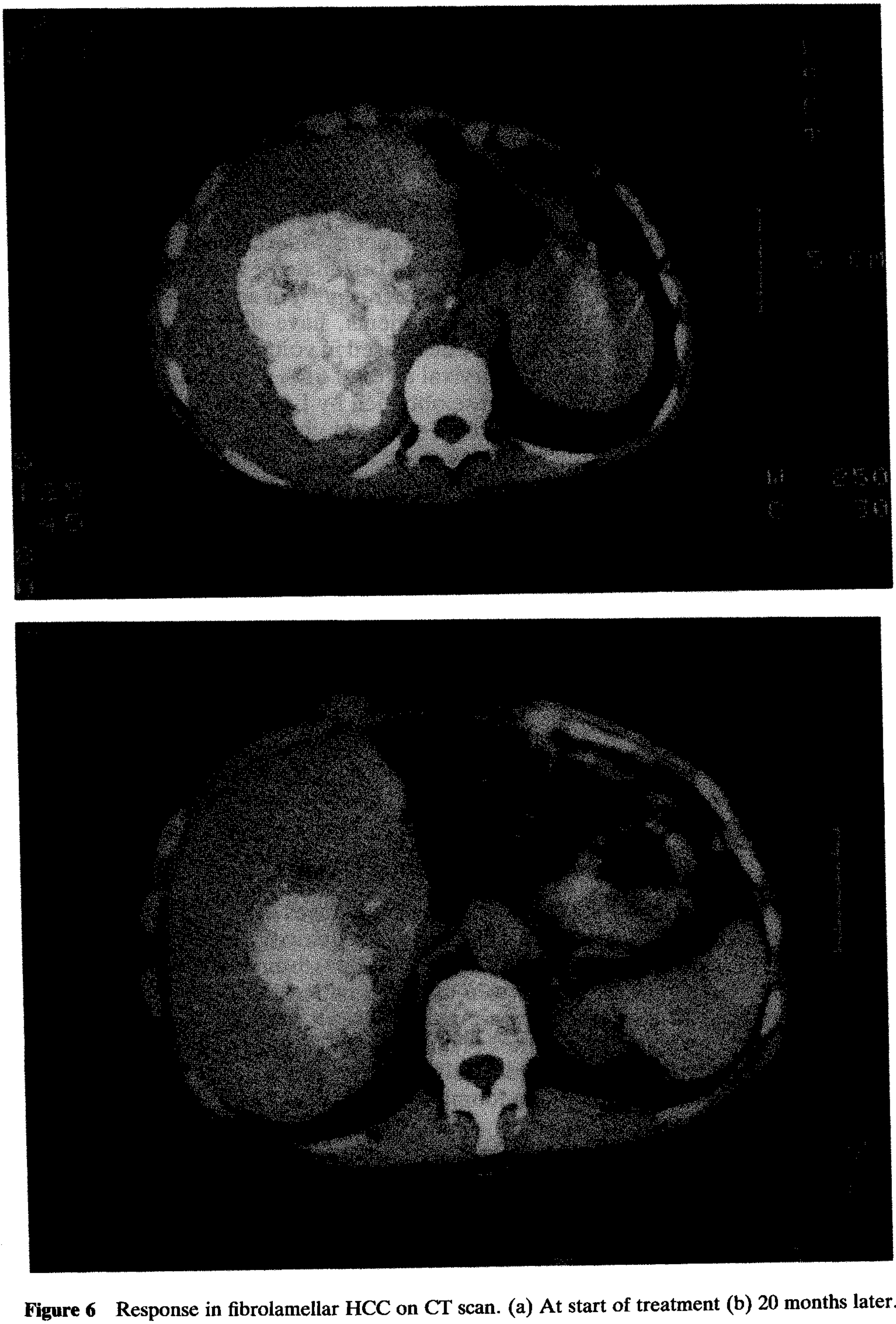

**Figure 6** Response in fibrolamellar HCC on CT scan. (a) At start of treatment (b) 20 months later.

arterial Lipiodol-epirubicin and systemic epirubicin treatment. Following $^{131}$I-Lipiodol therapy the tumour regressed to 20% of its original volume (Figure 6), and the patient remains asymptomatic 19 months later. Spence *et al.*[21] have reported long term survival in a similar case treated by Lipiodol-targeted chemotherapy; Lipiodol-targeted radiotherapy may also be effective in fibrolamellar HCC.

The mean survival in HCC was 7.1 (SD 6.0) months. Survival in Stage I was better than in Stage II, although the number of patients with Stage I disease was very small. The mean survival of patients showing partial response (11.6 months, SD 6.8) was significantly better than that of patients with tumour progression or no change (3.8 months, SD 1.7; $p = 0.004$, pooled estimate of variance). This accords with previous work on $^{131}$I brachytherapy: Order *et al.*[22], in an analysis of the results of targeted $^{131}$I antibody therapy in 343 patients, have demonstrated improved survival in those patients whose tumours decreased in volume by more than 30% on serial CT scanning. The cumulative survival rates of 61% at 6 months and 31% at 1 year in this series were identical to those obtained by Le Jeune *et al.*[10], and similar to the results of recent trials of Lipiodol-targeted chemotherapy[23,24]. The lower toxicity of $^{131}$I-Lipiodol radiotherapy may make it the treatment of choice for small (Stage I) tumours in older, cirrhotic patients who are at high risk of complications following surgical resection.

Multiple treatments might be administered more easily via an infusion pump than by repeated percutaneous cannulation as employed in this series. However, external pumps are cumbersome and frequently complicated by thrombosis and sepsis, whereas implantable systems are more convenient but are associated with a significant local complication rate. Sclerosing cholangitis, chemical hepatitis, gastritis and cholecystitis have all been reported[25]. The only treatment-related death in this series resulted from sepsis and hepatorenal failure. The mean radiation dose to the hepatic parenchyma for all cases was less than 4 Gy, and no evidence of hepatotoxicity was detected in the remaining patients. However, as a result of this lethal complication we have modified our protocol and now give a smaller amount of isotope in cirrhotic patients undergoing repeat treatments.

In summary, the results of $^{131}$-Lipiodol localisation in CCA were disappointing, with evidence of tumour targeting in only one patient. However, the low toxicity of $^{131}$I-Lipiodol might justify its use as part of a regime combining several treatment modalities. In HCC $^{131}$I-Lipiodol therapy has advantages over current non-surgical therapies in terms of cytotoxic potential and low toxicity, resulting in objective response in 40% of patients. A good result was also observed in one patient with fibrolamellar HCC. $^{131}$I-Lipiodol proved useful in patients with tumour recurrence following surgery: it may also have a possible role in treating intrahepatic recurrences following orthotopic liver transplant. Caution is advocated in patients with poor hepatic or renal function, particularly if treatments are repeated. We believe that there is a need for a larger randomised controlled study of this modality in patients with Stage I and II HCC.

### *References*

1. Nakakuma, K., Tashiro, S., Uemura, K. *et al.* (1979) An attempt for increasing effects of hepatic artery ligation for advanced hepatoma (English abstract). *Jap-Deutsche Med. Beriehte*, **24**, 675–682
2. Madsen, M., Park, C. and Thakur, M. (1988) Dosimetry of Iodine-131 ethiodol in the treatment of hepatoma. *J. Nucl. Med.*, **29**, 1038–1044

3. Raoul, J., Bourguet, P., Bretagne, J. *et al.* (1988) Hepatic artery injection of I-131-labelled Lipiodol. Part I; biodistribution study results in patients with hepatocellular carcinoma and liver metastases. *Radiology*, **168**, 541–545
4. Park, C.H., Suh, J.H., Yoo, H.S. *et al.* (1986) Evaluation of intrahepatic I-131 ethiodol on a patient with hepatocellular carcinoma; therapeutic feasibility study. *Clin. Nucl. Med.*, **11**, 514–517
5. Park, C.H., Suh, J.H., Yoo, H.S. *et al.* (1987) Treatment of hepatocellular carcinoma (HCC) with radiolabelled Lipiodol: a preliminary report. *Nucl. Med. Commun.*, **8**, 1075–1087
6. Raoul, J.L., Duvauferrier, R., Bourguet, P. *et al.* (1986) Angiography with 131-Iodine-labelled oidized oil in malignant hepatoma (English abstract). *J. Radiol.*, **67**, 797–801
7. Tsai, C., Kusumoto, Y., Harada, R. *et al.* (1986) Effect of intrahepatic arterial infusion of $^{131}$I-labelled Lipiodol on hepatocellular carcinoma of rat. *Ann. Acad. Med. Singapore*, **15**, 521–524
8. Bretagne, J., Raoul, J., Bourguet, P. *et al.* (1988) Hepatic artery injection of I-131-labelled Lipiodol. Part II; preliminary results of therapeutic use in patients with hepatocellular carcinoma and liver metastases. *Radiology*, **168**, 547–550
9. Park, C.H., Yoo, H.S. and Suh, J.H. (1990) Critical evaluation of I-131-Lipiodol therapy for hepatocellular carcinoma. *Eur. J. Nucl. Med.*, **16(Suppl)**, S143
10. Le Jeune, J., Bourguet, P., Victor, G., Therain, F., Lemaire, B. and Collet, H. (1990) I 131-Lipiodol in the treatment of hepatocellular carcinoma: Results of a multiceter phase II study of fifty patients. *Eur. J. Nucl. Med.*, **16(Suppl)**, S143
11. Hind, R., Perring, S., Fleming, J. Birch, S., Batty, V. and Taylor, I. (1991) Lipiodol is a potential vehicle for selective radiotherapy in liver metastases (abstract). *Br. J. Surg.*, **78**, 755–756
12. Novell, J.R., Dusheiko, G., Markham, N., Reddy, K., Dick, R. and Hobbs, K. (1991) Selective regional chemotherapy of unresectable hepatic tumours using Lipiodol. *HPB Surg.*, **4**, 223–236
13. Okuda, K., Ohtsuki, T., Obata, H. *et al.* (1985) Natural history of hepatocellular carcinoma and prognosis in relation to treatment. *Cancer*, **56**, 918–928
14. WHO handbook for reporting results of cancer treatment. (1979) Geneva: World Health Organisation
15. Sorenson. J.A. and Phelps, M.E. (1980) *Physics in nuclear medicine*. Orlando: Grune and Stratton
16. Eary, J.F., Appelbaum, F.L., Durack, L. and Brown, P. (1989) Preliminary validation of the opposing view method for quantitative gamma camera imaging. *Med. Phys.*, **16**, 382–387
17. Novell, J.R., Hilson, A. and Hobbs, K. (1991) Ablation of recurrent primary liver cancer using 131I-Lipiodol. *Postgrad. Med. J.*, **67**, 393–395
18. Nakajo, M., Kobayashi, H., Shimabukuro, K. *et al.* (1988) Biodistribution and *in vivo* kinetics of iodine-131 Lipiodol infused via the hepatic artery of patients with hepatic cancer. *J. Nucl. Med.*, **29**, 1066–1077
19. McAleer, J.J.A., Dickey, W., Clarke, R., Johnston, G.W. and Callender, M.E. (1987) Regional lipiodolized chemotherapy for cholangiocarcinoma associated with oral contraceptives. *Postgrad. Med. J.*, **63**, 583–584
20. Stillwagon, G.B., Order, S.E., Klein, J.L. *et al.* (1987) Multi-modality treatment of primary nonresectable intrahepatic cholangiocarcinoma with $^{131}$I anti-CEA; a radiation therapy oncology group study. *Int. J. Radiation Oncology Biol. Phys.*, **13**, 687–695
21. Spence, R.A.J., Rosen, A., Krige, J.E.J., Blumgart, R.L., Temple-Camp, C.R.E. and Terblanche, J. (1987) Unresectable fibrolamellar hepatocellular carcinoma treated with intra-arterial Lipiodolised doxorubicin. *S. Afr. Med. J.*, **72**, 701–703
22. Order, S.E., Klein, J.L., Leichner, P.K. and Stillwagon, G.B. (1991) New therapeutic approaches for the management of hepatocellular carcinoma in the United States. In: *Etiology, pathology and treatment of hepatocellular carcinoma in North America* (Advances in applied biotechnology series, Volume 13), eds. Tabor, E., Di Bisceglie, A.M., Purcell, R.H. Houston: Portfolio
23. Vetter, D., Wenger, J-J., Bergier, J-M., Doffoel, M. and Bockel, R. (1991) Transcatheter oily chemoembolization in the management of advanced hepatocellular carcinoma in cirrhosis: results of a Western comparative study in 60 patients. *Hepatology*, **13**, 427–437
24. Kanematsu, T., Furuta, T., Takenaka, K. *et al.* (1989) A 5 year experience of lipiodolization: selective regional chemotherapy for 200 patients with hepatocellular carcinoma. *Hepatology*, **10**, 98–102
25. Balch, C.M. and Levin, B. (1987) Regional and systemic chemotherapy for colorectal metastases to the liver. *World J. Surg.*, **11**, 521–526

*(Accepted by S. Bengmark 11 March 1993)*

### INVITED COMMENTARY

Following Nakakuma and his colleagues surgical observations on the selective retention of liver tumours[1], there has been increasing interest in utilising this "vehicle" for the delivery of both cytotoxic chemotherapy and radiotherapy. Initial results have been promising in terms of tumour shrinkage and symptomatic improvement but consistent survival data is lacking. This paper reports the preliminary results of a small pilot study of 131-I lipiodol in both cholangiocarcinoma and hepatocellular carcinoma.

There are several aspects which deserve comment. The method of delivery employed is by percutaneous catheterisation of the femoral artery. Other groups, including our own[2], have preferred operative placement of the catheter with a subcutaneous "port". This has the advantage of allowing repeated daily injections so enabling fractionated doses to be given at repeated intervals. In addition it reduces the rate of contamination and spillage of radioactive material. For example if spillage occurred in the X-ray department this would inevitably mean closure of a screening room for several weeks! In addition the risks of radioactive exposure to staff using this technique is greater than with a closed system.

With regard to tumour assessment the ratios of tumour: liver depends very much upon whether the liver region-of-interest was the liver or just a section of normal liver. If it was the former then to dose ratios of tumour: liver seem very high when judged by visual inspection of lipiodol images provided.

The lack of tumour-selective retention in cholangiocarcinoma is disappointing especially in view of the favourable uptake in hepatocellular carcinoma. The major effects however appear to be determined by the size of tumours. Multifocal small deposits being the most avid retainers of radioactivity. It should be noted that this is also a feature of liver metastases[2]. Clearly both pathological size and histology will need to be taken into account in determining selection of suitable patients.

The authors have paid particular attention to adverse effects which is a major consideration in phase I-II studies of this type. In general these are mild but hepatotoxicity is a theoretical possibility and accurate placement of the catheter is essential if gastritis and cholecystitis are to be avoided.

Overall this paper represents a valuable, albeit preliminary, evaluation of lipiodol localisation in primary liver tumours and suggests that further work on this novel treatment is justified.

Irving Taylor
Professor of Surgery and
Head of Department
University College
London

*References*

1. Nakakuma, K., Tashiro, S., Uemura, K. *et al.* (1979) An attempt for increasing effects of hepatic artery ligation for advanced hepatoma (English abstract). *Jap-Deutsche Med. Beriehte*, **24**, 675–682
2. Hind, R.E., Loizidou, M., Perring, S., Fleming, J. and Batty, V. (1992) Biodistribution of lipiodol following hepatic artery injection. *Brit. J. Surg.*, **79**, 952–954

